# Application of Natural Flavonoids to Impart Antioxidant and Antibacterial Activities to Polyamide Fiber for Health Care Applications

**DOI:** 10.3390/antiox8080301

**Published:** 2019-08-12

**Authors:** Ya-Dong Li, Jin-Ping Guan, Ren-Cheng Tang, Yi-Fan Qiao

**Affiliations:** 1 College of Textile and Clothing Engineering, Soochow University, 178 East Ganjiang Road, Suzhou 215021, China; 2 National Engineering Laboratory for Modern Silk, Soochow University, 199 Renai Road, Suzhou 215123, China

**Keywords:** antioxidant activity, antibacterial activity, flavonoids, polyamide fiber, adsorption

## Abstract

Polyamide fiber has the requirements for antioxidant and antibacterial properties when applied to produce functional textiles for heath care purposes. In this work, three natural flavonoids (baicalin, quercetin, and rutin) were used to simultaneously impart antioxidant and antibacterial functions to polyamide fiber using an adsorption technology. The relations of the chemical structures of flavonoids with their adsorption capability, adsorption mechanisms, and antioxidant and antibacterial activities were discussed. The Langmuir–Nernst adsorption model fitted the adsorption isotherms of the three flavonoids well. The adsorption kinetics of the three flavonoids conformed to the pseudo second-order kinetic model. Quercetin exhibited the highest affinity and adsorption capability, and imparted the highest antioxidant and antibacterial activities to polyamide fiber; and moreover, its antioxidant and antibacterial functions had good washing durability. This study demonstrates that the treatment using natural flavonoids is an effective way to exhance the health care functions of polyamide fiber.

## 1. Introduction

Health care textiles have attracted increasing attention in recent years. Textiles or apparel possessing antibacterial and antioxidant functions can offer health care effects. However, most textiles lack these two functions. Textile materials are easily infested by microbes. Microbial growth and proliferation on textiles can give rise to dermal infections, cross-infections, mildew formation, disease spread, allergic reactions, and foul odors [1]. As a consequence of the great importance of antibacterial properties, the antibacterial functionalization of textiles has attracted more and more interest. Up to now, the antioxidant properties of textiles have been less studied. Textiles containing antioxidants can function as a reservoir system steadily delivering antioxidants to the skin. When in contact with skin, such textiles have the ability to scavenge free radicals caused by skin degeneration, and protect skin tissues from oxidative stress and damage [2,3]. Antioxidant and antibacterial textiles can be utilized to prepare facial masks, patient clothes, and daily clothes for people who have skin diseases. Nowadays consumers are pursuing healthy and comfortable fiber materials which can provide and maintain an optimal microenvironment for healing some disorders or avoiding disease [4]. This promotes the development of novel healthcare textiles possessing antibacterial and antioxidant functions.

Polyamide fiber is one of the three major synthetic fibers, and its consumption is the second after polyester fiber. Polyamide 6 {poly[imino(1-oxohexane-1,6-diyl)]} and polyamide 6,6 {poly[N,N’-(hexane-1,6-diyl)adipamide]} are most extensively used in fiber and textile industries. Polyamide fiber possesses excellent performance such as good abrasion resistance, elastic resilience, mechanical properties, chemical resistance, temperature resistance, and processability [5], and has found wide applications in underwear, sports/leisure wear, and outerwear. In particular, polyamide fiber is often employed to manufacture socks, leggings, bras, knickers, tight sportswear, restrictive clothing, etc. As a result, these polyamide products have frequent contact with the skin when in use. In order to protect the skin and promote existential health, biological and cosmetic functions such as antioxidant and antibacterial activities as well as pleasant feeling, slimming, refreshing, skin glowing, anti-ageing, body care, fitness and health, etc. [6] are expected to be imparted to polyamide textiles.

Antibacterial polyamide fiber can be produced using two ways: the addition of inorganic antibacterial agents (e.g., silver and zinc oxide nanoparticles) into polyamide during fiber spinning [7,8], and the treatment of polyamide fiber using antibacterial agents in wet processing [9,10,11,12,13]. Because of the processing convenience, the latter is most often adopted. The antibacterial agents used in wet processing include cationic non-surfactant and surfactant agents (e.g., chlorhexidine, cetylpyridinium chloride) [9,10], chitosan [11], silver nanoparticles [12], and metal salts [13]. However, the aforementioned antibacterial agents cannot impart antioxidant properties to polyamide fiber. Plant extracts seem to be more preferred for imparting health care functions to textiles because of their non-toxicity, eco-friendliness, low irritation, and potential multi-functional properties [1,4]. In previous researches, the use of some natural dyes such as berberine, turmeric, madder, safflower yellow, and colors from walnut shells was found to confer good antibacterial functions to polyamide fiber [14,15,16,17,18]. Additionally, resveratrol as well as carotenoids from tomato processing wastes were used to treat polyamide fiber for enhanced antioxidant activity [2,19]. The action of the resveratrol treated polyamide fiber on the skin was assessed, and an improved antioxidant capacity of the skin was revealed [2].

**Figure 1:**
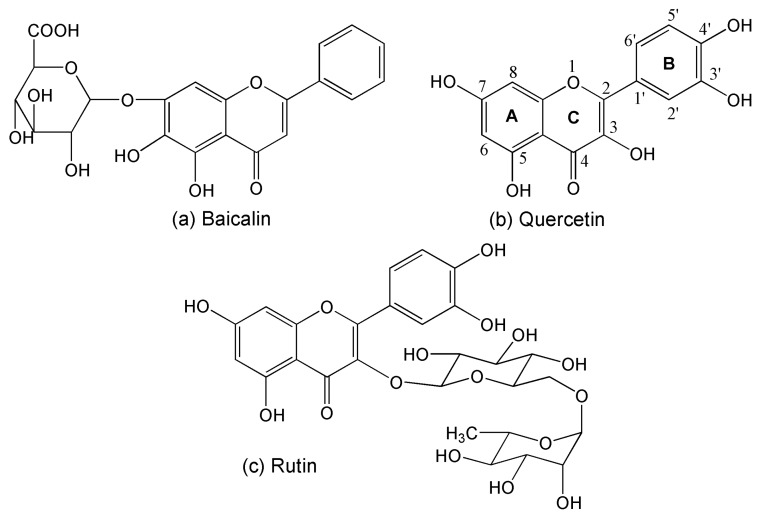
Chemical structures of the studied flavonoids.

In the light of the requirements for the functional properties of polyamide fiber for health care purposes, this study aims to use natural flavonoids as functional agents to treat polyamide fiber to simultaneously enhance its antioxidant and antibacterial activities. In this work, polyamide fiber was treated by means of an adsorption technique with three flavonoids (baicalin, quercetin, and rutin, whose chemical structures are depicted in Figure 1). The pH dependence of flavonoid adsorption was discussed, and the adsorption kinetics and isotherms as well as the adsorption mechanisms of flavonoids were studied. Furthermore, the antioxidant and antibacterial activities of treated polyamide fiber as well as their washing durability were evaluated.

## 2. Materials and Methods

### 2.1. Materials

A knitted polyamide fabric with a weight of 168.3 g/m^2^ was provided by Kunshan Teng Fei Underwear Technology Co. Ltd. (Kunshan, China). Baicalin with a purity of 85%, quercetin with a purity of 95%, and rutin with a purity of 95% were all purchased from Xi’an Qing Yue Biotechnology Co. Ltd. (Xi’an, China). 2,2′-Azino-bis(3-ethylbenzothiazoline-6-sulphonic acid) diammonium salt (ABTS) was purchased from Sigma–Aldrich Trading Co. Ltd. (Shanghai, China). Citric acid, hydrochloric acid, sodium hydroxide, disodium hydrogen phosphate, monopotassium phosphate, potassium persulfate, and potassium chloride were of analytical reagent grade. A commercial detergent was obtained from Shanghai Zhengzhang Laundering and Dyeing Co. Ltd. (Shanghai, China); the pH of 2 g/L detergent solution was about 6.5. Nutrient agar and nutrient broth were bought from Sinopharm Chemical Reagent Co. Ltd. (Shanghai, China) and Shanghai Sincere Biotech Co. Ltd. (Shanghai, China), respectively.

### 2.2. Flavonoids Adsorption and Polyamide Fabric Treatment

All the adsorption and treatment of flavonoids were carried out in sealed conical flasks placed in a XW-ZDR low-noise oscillated dyeing machine (Jingjiang Xinwang Dying and Finishing Machinery Factory, Jingjiang, China). The liquor ratio (the ratio of liquor volume to fabric weight) was 50:1, and the fabric weight was 2 g. A McIlvaine buffer consisting of citric acid and disodium hydrogen phosphate was added to adjust pH. At the end of the treatment process, the fabrics were rinsed in distilled water and then dried in the open air. In order to investigate the pH dependence of flavonoid adsorption, polyamide fabric was treated with 2% owf (on the weight of fabric) flavonoids whose pH values were adjusted within the range of 2.7 to 7.2; the temperature was started at 25 °C, and elevated to 70 °C at a heating rate of 2 °C/min, and subsequently the treatment was continued at 70 °C for 60 min. In order to study the adsorption rates of flavonoids, polyamide fabric was treated with 2% owf flavonoids at pH 2.79 and 70 °C for different times. The equilibrium adsorption isotherms for flavonoids on polyamide fabric were measured in a series of flavonoid solutions of various concentrations (1–12% owf) at pH 2.79 at 70 °C for 150 min; the isotherms were determined on the basis of the adsorption for 150 min as this time was enough for the equilibrium adsorption to be achieved. In order to determine the effect of the initial concentration of flavonoids on their uptake by polyamide fiber, polyamide fabric was treated with different concentration (2–10% owf) of flavonoids at pH 2.83; the temperature was started at 25 °C, and raised at a heating rate of 2 °C/min up to 70 °C with a holding time of 60 min. In adsorption researches, three parallel experiments were performed and their average results were used.

### 2.3. Measurements

#### 2.3.1. Uptake of Flavonoids

The absorption spectra and absorbance of flavonoid solutions were measured using a Shimadzu UV-1800 UV–vis spectrophotometer (Shimadzu Co., Kyoto, Japan). The percentage of flavonoid exhaustion was determined using a previously established absorbance/concentration relationship at the maximum absorption wavelength of flavonoid solutions using Equation (1):

(1) $$\mathrm{Exhaustion}\;(\%)\;=\frac{m_{0}-m_{1}}{m_{0}}\times 100$$

where *m*_0_ and *m*_1_ are the quantities of flavonoids before and after adsorption, respectively. The quantity of flavonoids on polyamide fabric was calculated by the difference in the initial and final concentrations of flavonoids in solution as well as the weight of the dried fabric.

#### 2.3.2. Zeta Potential

The zeta potential and isoelectric point of polyamide fabric were determined using the streaming potential method applied in a SurPASS electrokinetic analyzer (Anton Paar GmbH, Graz, Austria). A pair of fabric samples (ca. 10 × 20 mm^2^) was equilibrated in a 1 mM KCl supporting electrolyte solution at 20 °C. During the measurement, the electrolyte solution was forced through the packed fabric samples between two perforated Ag/AgCl electrodes in a measuring cell. The pH of solution was adjusted to the range of 3.2–8.5 with 0.1 M HCl and 0.1 M NaOH.

#### 2.3.3. Antioxidant Activity

The samples treated with 2–10% owf flavonoids in the section of building-up ability were used to evaluate the antioxidant activity. The antioxidant activity of polyamide fabric was measured using a previously reported ABTS radical cation decolorization assay [20]. ABTS was first dissolved in water to a 7 mM concentration, and then the ABTS stock solution was employed to react with 2.45 mM potassium persulfate (final concentration) so as to produce the ABTS radical cation (ABTS·^+^). The mixture was allowed to stand in the dark at room temperature for 12–16 h. Before use, the ABTS·^+^ solution was diluted with a phosphate buffer (0.1 M, pH 7.4) to reach an absorbance of 0.700 ± 0.025 at 734 nm, and afterwards 10 mg of fabric sample was immersed into 10 mL of ABTS·^+^ solution. After 30 min, the scavenging capability of ABTS·^+^ at 734 nm was calculated according to Equation (2):

(2) $$\mathrm{Antioxidant}\;\mathrm{activity}\;(\%)=\frac{A_{\mathrm{ctrl}}-A_{\mathrm{spl}}}{A_{\mathrm{ctrl}}}\times 100$$

where *A*_ctrl_ is the initial absorbance of the ABTS·^+^, and *A*_spl_ is the absorbance of the remaining ABTS·^+^ in the presence of fabric sample. The average of three tests for antioxidant activity was reported.

#### 2.3.4. Antibacterial Activity

The samples treated with 2% and 10% owf flavonoids in the section of building-up ability were used to evaluate the antibacterial activity. The test on the antibacterial activity of polyamide fabric against *Staphylococcus aureus* (*S. aureus*, ATCC 6538) and *Escherichia coli* (*E. coli*, ATCC 8099) was performed according to GB/T 20944.3-2008 [21]. In the test, standard cotton fabric and tested polyamide fabric were used. The fabric fragments (0.75 g) were dipped into the conical flasks with bacteria, which were oscillated in a shaker at a required temperature (24 °C for *S. aureus* and 30 °C for *E. coli*) for 24 h. After completion of vibration, the bacteria suspension was diluted 1000 times. Subsequently, the diluted bacteria solution was inoculated onto the agar plates which were stored at 37 °C for a desired time (48 h for *S. aureus* and 24 h for *E. coli*). In the end, the quantity of visual bacterial colonies was counted, and the antibacterial activity was calculated using Equation (3):

(3) $$\mathrm{Antibacterial}\;\mathrm{activity}\;(\%)=\frac{N_{\mathrm{ctrl}}-N_{\mathrm{spl}}}{N_{\mathrm{ctrl}}}\times 100$$

where *N*_ctrl_ and *N*_spl_ are the quantities of the visual bacterial colonies for standard cotton fabric and tested polyamide fabric, respectively. The average of three tests for antibacterial activity was reported.

#### 2.3.5. Durability of Antioxidant and Antibacterial Effects

The samples treated with 2% owf and 10% owf flavonoids were subjected to repeated laundrying. After repeated laundrying, the antioxidant and antibacterial activities were measured. The washing test was briefly described in the following: the samples were immersed into the washing solution containing 2 g/L commercial detergent using a liquor ratio of 50:1. Afterwards, the samples were stirred and left for 10 min at 40 ± 2 °C in a Wash Tec-P fastness tester (Roaches International, West Yorkshire, UK). After washing, the fabrics were gently squeezed and rinsed with distilled water. This treatment was repeated 1, 5, 10, 20 and 30 times.

## 3. Results and Discussion

### 3.1. Adsorption Characteristics of Flavonoids

#### 3.1.1. pH Dependence of Flavonoids Adsorption

**Figure 2:**
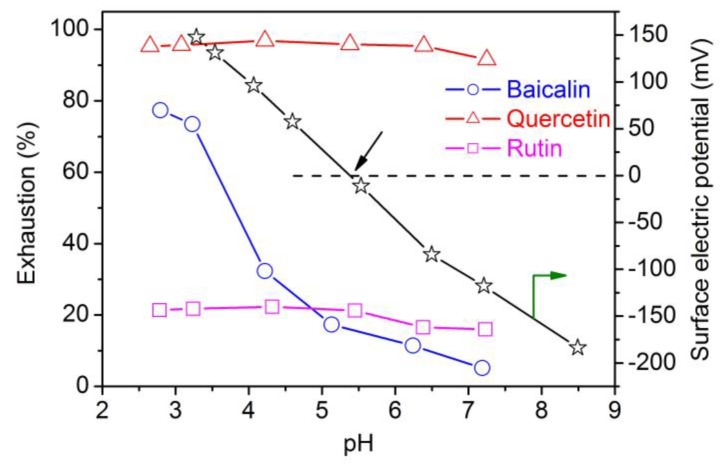
pH Dependence of flavonoids adsorption and surface electric potential of polyamide fiber.

Because pH can affect the surface potential of polyamide fiber, the ionization of functional groups in flavonoids, and the stability of flavonoids, it would have an impact on the adsorption of flavonoids. Thus, pH was considered as a significant factor having impact on the adsorption of flavonoids. The impact of pH on the uptake of flavonoids is depicted in Figure 2. From Figure 2, it can be interestingly observed that pH had different effects on the adsorption of three flavonoids. The uptake of baicalin by polyamide fiber was significantly dependent on pH. The uptake of baicalin was 77.4% at pH 2.79, and it dropped with an increase in pH. It implies that the adsorption of baicalin is primarily caused by the electrostatic attractions between anionic baicalin and protonated polyamide fiber. In order to explain the electrostatic attractions between baicalin and polyamide fiber having amphoteric character at different pHs, the surface electric potential of polyamide fiber was determined. Figure 2 shows that as pH increased, the surface electric potential had a shift from positive to negative. The zero point of net charge (isoelectric point) was at pH 5.63 or so. At a low pH, the protonation of amino groups in polyamide fiber is increased. Thus the electrostatic interactions between baicalin and polyamide fiber is enhanced, thereby resulting in the high adsorption of baicalin. The similar pH dependence of adsorption was also observed in our earlier study about the treatment of silk by baicalin [22].

Figure 2 shows that pH had a very small impact on the uptake of quercetin and rutin. The uptake of quercetin and rutin remained almost unchanged within pH 2.65–6.39 and 2.78–5.45, respectively. Quercetin and rutin had a slightly decreased uptake when pH exceeded 6.39 and 5.45, respectively, most likely due to their reduced stability at increasing pH. In addition, it is clear from Figure 2 that on the whole, quercetin showed the maximum adsorption on polyamide fiber, rutin displayed very poor adsorption ability, and baicalin had an adsorption extent between the adsorption of quercetin and rutin. The three flavonoids exhibited rather different pH dependence and capability of adsorption. These results are likely indicative of different adsorption mechanisms of three flavonoids.

#### 3.1.2. Adsorption Kinetics of Flavonoids

**Figure 3:**
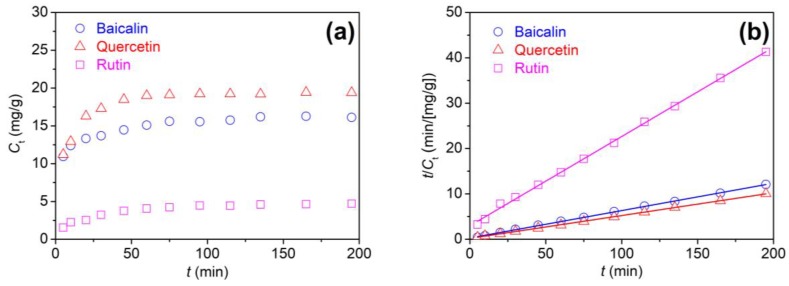
Adsorption rates of three flavonoids for polyamide fiber: (**a**) *C*_t_~*t* and (**b**) *t*/*C*_t_~t.

The adsorption kinetics is vital for controlling the efficiency and uniformity of adsorption process. The adsorption kinetics of flavonoids was studied through the relationships between their adsorption amount (*C*_t_) and time (*t*). As depicted in Figure 3a, the adsorption rates of three flavonoids on polyamide fiber were not fast because flavonoids were applied at a moderate temperature (70 °C). The adsorption quantity of three flavonoids increased gradually with prolonged time. After about 100 min, the adsorption quantity of flavonoids remained almost unchanged. It indicates that the adsorption equilibrium was achieved. It seemed that the adsorption rate of quercetin was the fastest while that of rutin was the slowest.

To compare the adsorption behaviors of three flavonoids, the pseudo second-order kinetic model was utilized to simulate the experimental data. This model can be expressed using Equation (4) [23]:

(4) $$\frac{t}{C_{t}}=\frac{1}{kC_{e}^{2}}+\frac{1}{C_{e}}t$$

where *k* denotes the rate constant of adsorption, and *C_t_* and *C_e_* denote the adsorption quantity of flavonoids at time *t* and at equilibrium.

If the adsorption conforms to the aforementioned model, *t*/*C_t_* and *t* have a linear relation. Figure 3b displays the linear relation of the *t*/*C_t_*~*t* plot. By regression analysis, the fitted straight line was got. Its slope and intercept were utilized to calculate *k* and *C_e_*. Additionally, based on this model, the half adsorption time (*t*_1/2_) and initial adsorption rate (*r_i_*) were estimated according to Equations (5) and (6), respectively:

(5) $$t_{1/2}=\frac{1}{kC_{e}}$$

(6) $$r_{i}=kC_{e}^{2}$$

Table 1 shows the correlation coefficients (*R*^2^) of this model. The *R*^2^ values were higher than 0.998 for three flavonoids. It indicates that this model is applicable to describe the adsorption rates of flavonoids for polyamide fiber. Table 1 shows the great differences in the kinetic parameters between three flavonoids. Quercetin exhibited the highest initial adsorption rate and the shortest half adsorption time with the moderate adsorption rate constant. Baicalin also had a short half adsorption time and a high initial adsorption rate. Rutin displayed a very low initial adsorption rate and a very long half adsorption time. Moreover, the equilibrium adsorption of flavonoids increased in the following order: rutin < baicalin < quercetin. These observations indicate that quercetin would have the highest affinity to polyamide fiber whereas the affinity of rutin is the lowest.

#### 3.1.3. Adsorption Isotherms of Flavonoids

**Figure 4:**
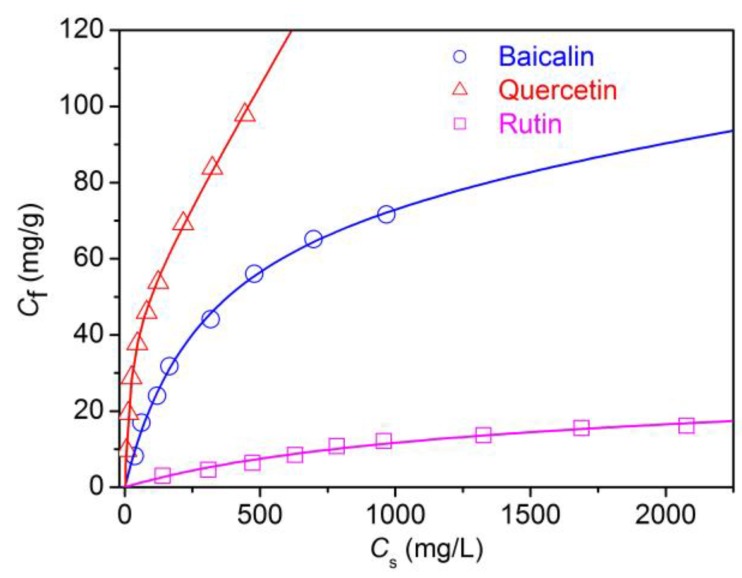
Adsorption isotherms of flavonoids for polyamide fiber and Langmuir–Nernst plots.

The research on the equilibrium adsorption isotherms is used to explore the adsorption mechanisms of flavonoids on polyamide fiber, and help to discuss the interactions between flavonoids and polyamide fiber. The adsorption isotherms of three flavonoids on polyamide fiber are depicted in Figure 4. Three equilibrium adsorption equations (Langmuir, Freundlich and Langmuir–Nernst) were utilized to simulate the experimental data.

The Langmuir isotherm is expressed using the following equation:

(7) $$C_{f}=\frac{SK_{L}C_{s}}{1+K_{L}C_{s}}$$

where *C_f_* (mg/g) and *C_s_* (mg/L) denote the concentration of flavonoids on polyamide fiber and in solution at equilibrium, respectively; *S* denotes the adsorption saturation of flavonoids on polyamide fiber; *K_L_* denotes the Langmuir affinity constant.

The Freundlich isotherm is expressed using the following equation:

(8) $$C_{f}=K_{F}C_{s}^{n}$$

where *K_F_* denotes the Freundlich affinity constant, and *n* reflects adsorption intensity or surface heterogeneity.

The dual Langmuir–Nernst isotherm is expressed using the following equation:

(9) $$C_{f}=C_{P}+C_{L}=K_{P}C_{S}+\frac{SK_{L}C_{s}}{1+K_{L}C_{s}}$$

where *C_P_* and *C_L_* denote the concentration of flavonoids on polyamide fiber following Nernst type partitioning and Langmuir adsorption mechanisms, respectively; *S* denotes the saturation adsorption of flavonoids on polyamide fiber by Langmuir mechanism; *K_P_* and *K_L_* denote the partition coefficient and the Langmuir affinity constant, respectively.

The experimental data in Figure 4 were fitted using a nonlinear least-squares fitting approach. To assess the fitting degree, the normalized deviation (*ND*) of the experimental data was determined using the following equation:

(10) $$ND(\%)=100\times \frac{1}{N}\sum_{i=1}^{N}(\frac{C_{f,\exp,i}-C_{f,\mathrm{calc},i}}{C_{f,\exp,i}})$$

where *C*_f,exp,i_ and *C*_f,calc,i_ denote the experimental and calculated amount of flavonoids adsorption by polyamide fiber, respectively; the index “*i*” denotes to the serial number of data points; *N* denotes the sum of data points.

Table 2 lists the *ND* values of the experimental data. Overall, the Langmuir–Nernst model had the lowest *ND*, and gave the best fitting to the experimental points. The Langmuir–Nernst curves of Figure 4 almost passed all the experimental points precisely. These findings suggest that the Langmuir–Nernst model is the most suitable to characterize the adsorption performance of flavonoids on polyamide fiber. According to this model, the electrostatic interaction operating between flavonoids and polyamide fiber is responsible for Langmuir adsorption, whereas the non-electrostatic interactions are responsible for Nernst adsorption. In this work, the adsorption isotherm measurement was carried out at pH 2.79 which was below the isoelectric point (5.63) of polyamide fiber. At this pH, the protonation extent of amino groups in polyamide fiber is great. The protonated amino groups in polyamide fiber can adsorb the negatively charged flavonoids through electrostatic interaction, contributing to Langmuir adsorption.

Additionally, in some previous investigations, macroporous polymer containing amino groups exhibited high adsorption ability towards phenolic compounds because of hydrogen bonding or acid-base interactions between them [24,25]. In our previous research, we also found that the electrostatic and hydrogen bond interactions between tea polyphenols and polyamide fiber contributed to Langmuir adsorption [26]. These previous findings suggest that in addition to electrostatic interaction, the hydrogen bond between flavonoids and polyamide fiber also can contribute to Langmuir adsorption. Flavonoids have abundant hydroxyl groups. Polyamide fiber contains abundant amide groups as well as a small amount of amino and carboxyl groups. Thus, flavonoids interact with polyamide fiber through hydrogen bond.

For the adsorption of baicalin, the electrostatic interaction between baicalin and polyamide fiber is readily explained. The dissociation constant (p*K*_a_) of 7-glucuronic acid in A ring is 5.05 [27]. The p*K*_a_ values of 6-OH and 5-OH in A ring are 7.6 and 10.1, respectively [28]. At pH 5.05, the ionization degree of carboxyl groups is 50%. At pH 2.79 set in the present study, the partially ionized carboxyl groups in baicalin interact with the protonated amino groups in polyamide fiber through electrostatic attraction. After these ionized baicalin molecules are adsorbed by polyamide fiber, the ionization balance of 7-glucuronic acid is broken, which accelerates the further dissociation of 7-glucuronic acid. Thus, baicalin can continue to be adsorbed by polyamide fiber. Carboxyl groups have higher dissociation degree than hydroxyl groups. Therefore, it is reasonable to conclude that the electrostatic interaction between the ionized carboxyl groups in baicalin and the protonated amino groups in polyamide fiber is responsible for Langmuir adsorption.

Quercetin and rutin have no carboxyl groups in their structures. During the adsorption process their ion-ion interactions with polyamide fiber are associated with the deprotonated phenolic hydroxyl groups in their structures. The dissociation constants of quercetin obtained from different literatures showed significant variation. The list of the reported values of p*K*_a1_ gives p*K*_a1_ = 5.7, 6.6, 6.7, 7.03, 7.4, 7.7, 8.2, and 9.0 [29,30]. In general, the acidity of OH groups in different substitution sites decreases in the order: 7-OH > 4′-OH > 3-OH. It is supposed that after a small amount of hydroxyl groups in quercetin are deprotonated, they can be adsorbed by polyamide fiber through electrostatic interaction. Thus, the dynamic ionization equilibrium of quercetin is broken which facilitates the further disassociation of quercetin and the subsequent adsorption of quercetin on polyamide fiber. The dissociation constant (p*K*_a1_) of rutin is 7.1 [31]. The ion-ion interactions of rutin with polyamide fiber are similar to those of quercetin.

In addition to ion-ion interaction and hydrogen bonding, there exist van der Waals attraction and hydrophobic interactions between flavonoids and polyamide molecules. These interactions can be responsible for Nernst adsorption. Polyamide fiber contains considerable methylene groups, which have interactions with the aromatic moieties of flavonoids through hydrophobic and non-polar der Waals forces.

Table 3 lists the adsorption parameters for the Langmuir–Nernst isotherms. Baicalin exhibited the highest saturation adsorption, and much lower *K*_L_ and *K*_P_ values than quercetin, due to the fact that carboxyl groups in baicalin have higher dissociation degree than hydroxyl groups in quercetin. Quercetin displayed relatively high saturation adsorption, and the highest *K*_L_ and *K*_P_ values. This indicates that quercetin has the highest affinity to polyamide fiber as compared with baicalin and rutin. Moreover, the Nernst adsorption caused by hydrophobic interaction and non-polar van der Waals force has an important contribution to total adsorption. Therefore, it is not difficult to understand why quercetin exhibits the fastest adsorption and high adsorption quantity as aforementioned.

It is worth noting that the *K*_L_ and *K*_P_ values as well as the adsorption quantity of rutin are remarkably lower than those of quercetin. Rutin and quercetin have similar chemical structures. Their only difference is that rutin has a glycosidic linkage at position 3. The presence of a glycosidic moiety not only increases molecular weight and size, but also causes a steric hindrance. Both these factors exert a negative impact on the diffusion of rutin into the interior of polyamide fiber whose physical structure is compact, and accordingly decrease the adsorption quantity of rutin.

#### 3.1.4. Initial Concentration Dependence of Flavonoids Adsorption

The function relation of the adsorption quantity and initial concentration of flavonoids reflects the building-up property which is very important for industrial application [32]. The flavonoids possessing excellent building-up performance can draw attention of manufacturers due to the advantage of eco-friendliness [33], high utilization, and sufficient functionalities. Hence, taking the practical application conditions in consideration, the test on the building-up properties of three flavonoids onto polyamide fiber was performed using a heating and holding approach.

**Figure 5:**
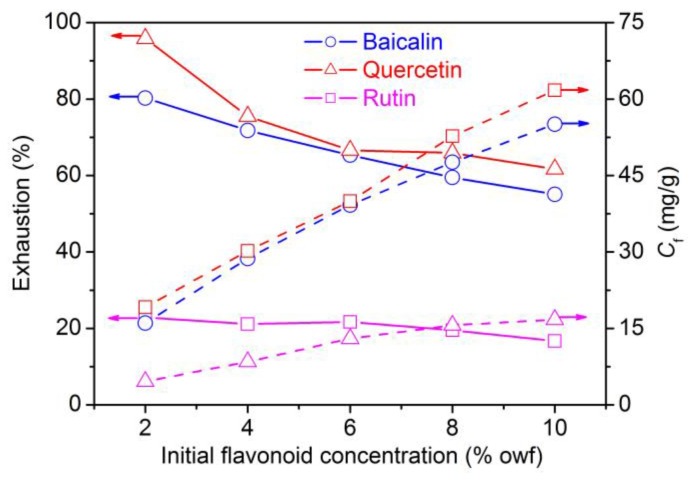
Uptake of flavonoids by polyamide fiber at different initial concentrations.

The adsorption quantity (*C*_f_) and exhaustion of flavonoids onto polyamide fiber are shown in Figure 5. In the case of baicalin and quercetin, the extent of adsorption almost increased linearly with increasing flavonoid dosage from 2% to 10% owf, and what is more, both of them kept high uptake at high dosages, indicating their great building-up ability towards polyamide fiber. On the contrary, rutin displayed very low exhaustion and adsorption quantity, indicating its poor building-up property and low utilization rate which would give rise to a high application cost.

### 3.2. Antioxidant and Antibacterial Properties of Flavonoids Treated Polyamide Fiber

#### 3.2.1. Antioxidant Property

The antioxidant properties of flavonoids have already been well discussed [30,31,34,35]. They are mainly associated with the substitution positions and total number of hydroxyl groups [35]. In general, the relationships between the antioxidant activity and structure of flavonoids are as follows [35,36]: the B-ring hydroxyl configuration of flavonoids is the most important factor in deciding radical scavenging ability due to its donation of hydrogen and an electron to radicals; a 3′,4′-catechol in the B-ring has great relevance to increased radical scavenging ability; flavonoid heterocycle (C-ring) is responsible for radical scavenging ability due to the conjugate effect of aromatic ring and 3-OH, whereas the substitution of 3-OH by glycosyl moiety inhibits radical scavenging; in comparison with the hydroxylation of B-ring, the influence of substituent groups in A-ring on radical scavenging is small. According to these above rules and by comparison of the chemical structures of the three flavonoids (Figure 1), it seems that the antioxidant activity of three flavonoids decreases in the following order: quercetin > rutin > baicalin. The aforementioned antioxidant activity of flavonoids mainly refers to foods and medical care. However, the antioxidant activity of flavonoids on polymeric fiber has been less studied and reported.

**Figure 6:**
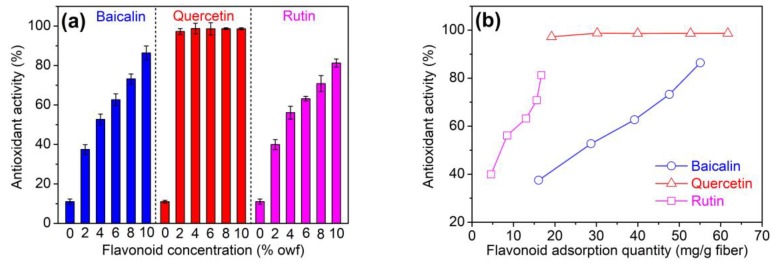
Antioxidant property of polyamide fibers treated using flavonoids at different dosages (**a**) and adsorption quantities (**b**).

Figure 6a shows the antioxidant property of polyamide fibers treated using three flavonoids at different concentrations (2–10% owf). As can be seen in Figure 6a, pristine polyamide fiber had a low antioxidant activity value of about 10%. After the treatment using three flavonoids, polyamide fiber displayed significantly improved antioxidant property. Moreover, the antioxidant property of polyamide fiber increased with increasing initial dosages of baicalin and rutin, whereas it always kept a very high level at different dosages of quercetin. As the dosage of three flavonoids reached 10% owf, the antioxidant activity of all the samples exceeded 80%.

At a dosage of 2% owf flavonoids, the antioxidant activity of polyamide fiber was 37.5% for baicalin, 97.2% for quercetin, and 39.9% for rutin, respectively. At this flavonoid dosage, the adsorption quantity of baicalin, quercetin, rutin on polyamide fiber was 16.06, 19.18, and 4.58 mg/g, respectively (Figure 5 and Figure 6b). Figure 6b reveals the relation between the antioxidant property and adsorption amount of three flavonoids. In the case that the adsorption amount was virtually at the same level, quercetin imparted the highest antioxidant activity to polyamide fiber, rutin was the second efficient although its adsorption was low, and baicalin had the lowest antioxidant activity. The great antioxidant ability of quercetin originates from its catechol structure of B-ring and multiple hydroxyl groups. The poor antioxidant ability of baicalin is due to the lack of catechol structure and 3-OH. Although rutin has a low adsorption extent, it bears the catechol structure and four phenolic hydroxyl groups and accordingly has better antioxidant property than baicalin.

#### 3.2.2. Antibacterial Property

It has been reported that baicalin, quercetin and rutin are potential antibacterial agents [37,38,39]. Some natural dyes containing baicalin, quercetin and rutin were able to improve the antibacterial properties of silk and wool fibers [40,41,42]. The antibacterial activity of natural dyes is usually considered to be mainly related to phenolic hydroxyl groups in their structures. In this study, three flavonoids were also expected to impart antibacterial activity to polyamide fiber.

**Figure 7:**
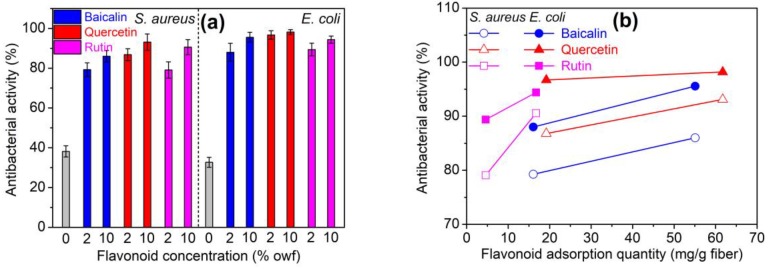
Antibacterial property of polyamide fibers treated using flavonoids at different dosages (**a**) and adsorption quantities (**b**).

Figure 7a displays the antibacterial property of polyamide fibers treated using baicalin, quercetin, and rutin at different concentrations. Pristine polyamide fiber showed poor antibacterial properties. Its antibacterial rate was 38.2% for *S. aureus* and 32.7% for *E. coli*. However, the treated fibers, especially the ones treated with quercetin, exhibited excellent antibacterial property. Moreover, the antibacterial activity increased with increasing initial dosages of flavonoids. At 2% owf flavonoids, the fibers treated using baicalin, quercetin and rutin exhibited good antibacterial property with an inhibition rate of 79.3%, 86.8%, and 79.1% against *S. aureus*, respectively, and 88.0%, 96.7%, and 89.4% against *E. coli*, respectively. Obviously, *S. aureus* is more tolerant to the treated polyamide fiber than *E. coli*. Figure 7b reveals the relation between the antibacterial property and adsorption amount of three flavonoids. At almost the same flavonoid adsorption amount, quercetin and rutin provided better antibacterial function than baicalin. This order of antibacterial activity is similar to that of antioxidant activity discussed above. But the difference of antibacterial activity among three flavonoids is evidently smaller than that of antioxidant activity.

#### 3.2.3. Laundering Durability of Antioxidant and Antibacterial Properties

**Figure 8:**
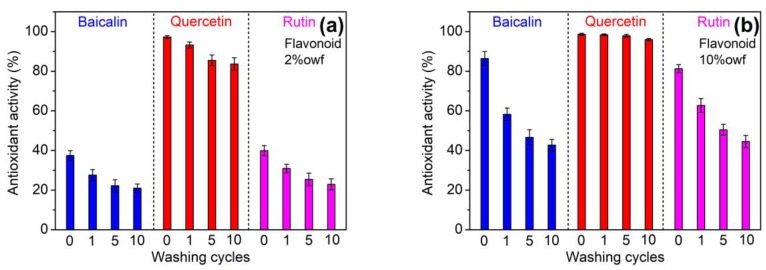
Antioxidant properties of polyamide fibers treated using 2% owf (**a**) and 10% owf (**b**) flavonoids after repeated laundering.

**Figure 9:**
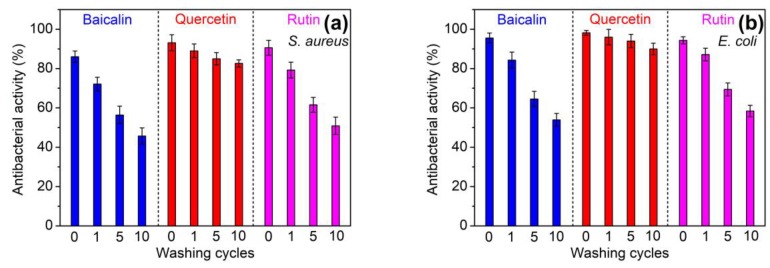
Antibacterial properties against *S. aureus* (**a**) and *E. coli* (**b**) of polyamide fibers treated using 10% owf flavonoids after repeated laundering.

Polyamide fiber is frequently subjected to repeated laundering when in use. Therefore, the laundering durability of antioxidant and antibacterial activities imparted to polyamide fiber is very important. Figure 8 displays the antioxidant activity of polyamide fibers treated with 2% and 10% owf flavonoids after 1, 5 and 10 cycles of washing. At two dosages of flavonoids, the antioxidant property of all the fibers declined gradually as the washing cycle increased. The reduction in antioxidant activity was the lowest for quercetin, whereas that was the highest for baicalin. Figure 9 displays the antibacterial property of polyamide fibers treated using 10% owf flavonoids after 1, 5 and 10 cycles of washing. As compared with antioxidant activity, antibacterial activity displayed the similar decrease tendency as the washing cycle increased. As pointed out in the adsorption isotherm section, quercetin has the highest affinity to polyamide fiber, and hence its desorption degree should be the lowest in the washing process of the treated polyamide fiber. Thus, quercetin can still provide very high antioxidant and antibacterial properties after repeated washings. For baicalin, its high decrease in antioxidant and antibacterial properties might be associated with its higher water solubility than that of quertin and rutin.

**Figure 10:**
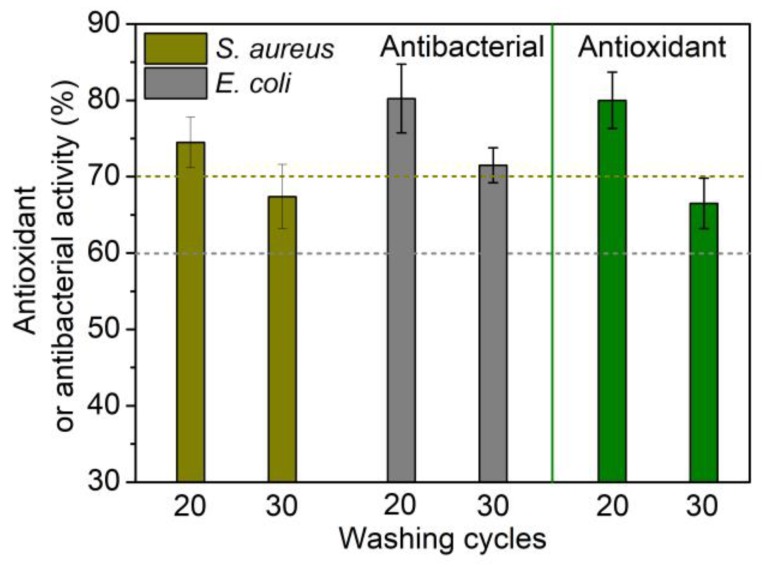
Antioxidant and antibacterial properties of polyamide fiber treated using 10% owf quercetin after 20 and 30 cycles of laundering.

Because quercetin exhibited high adsorption, excellent functions and good durability, the polyamide fabric treated using 10% quercetin was subjected to more repeated washings. Figure 10 displays that after 30 washings, the treated sample had an antioxidant activity of above 65%. After 20 washings, the antibacterial rate for both *S. aureus* and *E. coli* was higher than 70%. After 30 washings, the antibacterial rate for *S. aureus* was lower than 70%. According to GB/T 20944.3–2008 [21], the antibacterial textiles are required to have an antibacterial rate of above 70% for *S. aureus*, and above 60% for *E. coli*. Therefore the polyamide fabric treated using 10% quercetin can be resistant to 20 cycles of washing.

## 4. Conclusions

In the present study, three natural flavonoids were employed to simultaneously impart antioxidant and antibacterial activities to polyamide fiber using an adsorption technology. The adsorption of baicalin on polyamide fiber greatly depended on the pH of its solutions. The adsorption of the three flavonoids conformed to the pseudo second-order kinetic model. The adsorption isotherms of three flavonoids fitted the Langmuir–Nernst model. Quercetin showed the highest affinity to polyamide fiber and adsorption quantity, followed by baicalin, whereas rutin displayed poor adsorption capability due to the presence of a glycosidic moiety. Quercetin imparted very high antioxidant and antibacterial activities to polyamide fiber as compared with baicalin and rutin, and these functions displayed good resistance to washing. Although rutin provided slightly higher antioxidant and antibacterial activities than baicalin, and its low adsorption quantity would increase the processing cost of polyamide fabric, hence limiting its application. The present study suggests that the simultaneous antioxidant and antibacterial functionalization of polyamide fiber can be realized by treatments using natural flavonoids.

## Figures and Tables

**Table 1:** Kinetic parameters for flavonoid adsorption.

Flavonoid	*r*_i_ (mg/[g·min])	*t*_1/2_ (min)	*k* (×10^−2^g/[mg·min])	*C*_e_ (mg/g)	*R* ^2^
Baicalin	3.43	4.826	1.251	16.56	0.9995
Quercetin	5.03	3.954	1.272	19.88	0.9998
Rutin	0.34	15.100	1.303	5.08	0.9987

**Table 2:** Fitting degree of three equilibrium adsorption models.

Flavonoid	*ND* (%)		
	Langmuir	Freundlich	Langmuir–Nernst
Baicalin	4.59	12.81	5.04
Quercetin	15.76	7.55	3.13
Rutin	7.13	9.07	6.64

**Table 3:** Parameters for the Langmuir–Nernst isotherm of flavonoids adsorption.

Flavonoid	*S* (mg/g)	*K*_L_ (10^−3^ L/mg)	*K*_P_ (10^−3^ L/mg)
Baicalin	82.43	3.39	9.23
Quercetin	46.48	46.53	121.67
Rutin	22.10	0.91	1.13
